# Neurobiology of Wild and Hatchery-Reared Atlantic Salmon: How Nurture Drives Neuroplasticity

**DOI:** 10.3389/fnbeh.2018.00210

**Published:** 2018-09-11

**Authors:** Daan Mes, Kristine von Krogh, Marnix Gorissen, Ian Mayer, Marco A. Vindas

**Affiliations:** ^1^Department of Production Animal Clinical Sciences, Norwegian University of Life Sciences, Oslo, Norway; ^2^Department of Basic Sciences and Aquatic Medicine, Norwegian University of Life Sciences, Oslo, Norway; ^3^Department of Animal Ecology and Physiology, Institute of Water and Wetland Research, Radboud University, Nijmegen, Netherlands; ^4^Uni Environment, Uni Research AS, Bergen, Norway; ^5^Department of Neurobiology and Physiology, University of Gothenburg, Gothenburg, Sweden; ^6^Department of Food Safety and Infection Biology, Norwegian University of Life Sciences, Oslo, Norway

**Keywords:** *cfos*, *bdnf*, Atlantic salmon, immediate early gene, fish stocking, neuroplasticity, *in situ* hybridization

## Abstract

Life experiences in the rearing environment shape the neural and behavioral plasticity of animals. In fish stocking practices, the hatchery environment is relatively stimulus-deprived and does not optimally prepare fish for release into the wild. While the behavioral differences between wild and hatchery-reared fish have been examined to some extent, few studies have compared neurobiological characteristics between wild and hatchery-reared individuals. Here, we compare the expression of immediate early gene *cfos* and neuroplasticity marker brain-derived neurotrophic factor (*bdnf*) in telencephalic subregions associated with processing of stimuli in wild and hatchery-reared Atlantic salmon at basal and 30 min post (acute) stress conditions. Using *in situ* hybridization, we found that the expression level of these markers is highly specific per neuronal region and affected by both the origin of the fish, and exposure to acute stress. Expression of *cfos* was increased by stress in all brain regions and *cfos* was more highly expressed in the Dlv (functional equivalent to the mammalian hippocampus) of hatchery-reared compared to wild fish. Expression of *bdnf* was higher overall in hatchery fish, while acute stress upregulated *bdnf* in the Dm (functional equivalent to the mammalian amygdala) of wild, but not hatchery individuals. Our findings demonstrate that the hatchery environment affects neuroplasticity and neural activation in brain regions that are important for learning processes and stress reactivity, providing a neuronal foundation for the behavioral differences observed between wild and hatchery-reared fish.

## Introduction

Wild Atlantic salmon (*Salmo salar* L.) populations are declining worldwide (Parrish et al., 1998). Even in Norway – traditionally home to some of the healthiest Atlantic salmon stocks in the world – the number of wild salmon has more than halved in the last three decades (Thorstad and Forseth, 2015). Habitat degradation is one of the main reasons for salmon decline, and habitat restoration should thus be considered first and foremost as a conservation tool (Araki and Schmid, 2010). However, since habitat restoration is a slow and costly process, more immediate measures to support declining population numbers are frequently employed, such as the annual release of millions of hatchery-reared salmon into rivers worldwide through stocking programs (e.g., Palmé et al., 2012; Maynard and Trial, 2013). To this end, mature local salmon are captured and cross-fertilized, after which their offspring are reared in captivity and released in the wild at different developmental stages, ranging from eggs to juveniles but mostly at the smolt stage (Jonsson and Jonsson, 2009; Maynard and Trial, 2013). The hatchery environment provides optimal conditions for growth, which consequently leads to higher growth rates and larger body size at time of release for hatchery-reared fish compared to wild fish of the same age (Jonsson and Jonsson, 2009). However, cultured fish are generally reared under unnaturally high densities in stimulus-poor conditions, which leads to diminished behavioral plasticity in critical life skills such as antipredator and foraging behavior (Olla et al., 1998; Huntingford, 2004; Jonsson and Jonsson, 2009). For example, after release in the wild, stocked salmon often show reduced stomach fullness (Johnson et al., 1996) or ingestion of indigestible particles such as small rocks and plant material (Munakata et al., 2000). Behavioral deficits such as these contribute to lower post-release survival rates of stocked fish compared to their wild conspecifics (Johnson et al., 1996; Jonsson and Jonsson, 2009; Thorstad et al., 2011), raising both financial and ethical concerns for current stocking practices.

To increase the efficacy of stocking programs, research efforts are directed toward improving behavioral responses to stimuli from the natural environment and, ultimately, the fitness of hatchery-reared fish, through implementation of hatchery innovations such as environmental enrichment (reviewed by Johnsson et al., 2014), predator conditioning (reviewed by Brown et al., 2013), or foraging training (reviewed by Olla et al., 1998). In order to rear more “wild-like” fish under hatchery conditions, it is important to first understand how the neurobiological, physiological and behavioral characteristics of hatchery-reared fish differ from those of their wild conspecifics. While behavioral differences between wild and hatchery-reared fish have been described in several studies (e.g., Olla et al., 1998; Huntingford, 2004), the brain – the organ that underlies these behavioral differences – has remained much understudied. Environmental stimuli trigger and reinforce neuronal circuits through mobilization of neuropeptides such as brain-derived neurotrophic factor (*bdnf*), which promotes neurogenesis, cell survival and synaptic plasticity, thus altering the wiring of the brain in response to the rearing environment (Mattson et al., 2004; Ebbesson and Braithwaite, 2012; Shors et al., 2012; Gray et al., 2013). This process of brain modification due to environmental inputs is known as neuroplasticity, and reinforcement of neuronal circuits in response to experiences from the rearing environment affects how these neuronal circuits are activated by future stimuli, thus driving the fish’s behavior (Ebbesson and Braithwaite, 2012; Shors et al., 2012). Activation of neuronal circuits can be mapped through visualization of immediate early genes (IEGs) such as *cfos*, which is highly expressed after a neuron is activated, enabling us to take a snapshot of neuronal activation patterns in response to a stimulus such as acute stress (Okuno, 2011; Pavlidis et al., 2015). Thus, *cfos* and *bdnf* transcripts are established markers for neural activity and neuroplasticity, respectively, and they are important tools to help understand how the rearing environment affects the neurobiology of animals.

**FIGURE 1:**
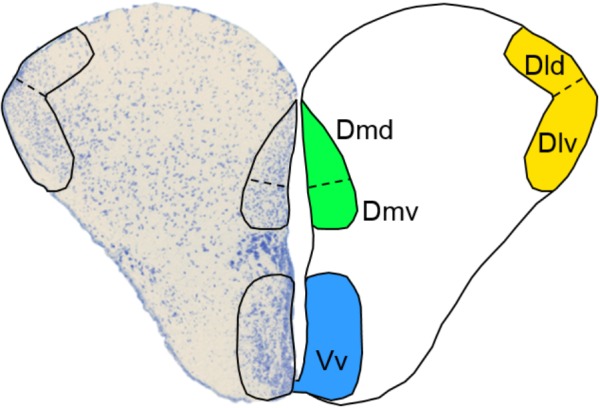
Selected subregions of the telencephalon. A transverse view of the Atlantic salmon telencephalon with a Toluidine Blue-stained left lobe and a schematic representation of the right lobe depicting the location of the dorsal (Dld) and ventral (Dlv) dorsolateral pallium, the dorsal (Dmd), and ventral (Dmv) dorsomedial pallium and the ventral part of the ventral telencephalon (Vv).

In vertebrates, cognitive processing is mainly under forebrain control. Therefore, it is imperative that we obtain a better understanding of how the rearing environment shapes forebrain functionality in order to improve fish quality in stocking programs. In contrast to mammals, teleost fish do not possess a cerebral cortex. However, fish telencephalic areas have been found to be functionally equivalent to mammalian forebrain regions and fish are capable of displaying complex behaviors including social decision making and associative learning, which are under forebrain regulation (Vargas et al., 2009; Kalueff et al., 2012; Stewart and Kalueff, 2012; Bshary and Brown, 2014). Within the telencephalon, the dorsolateral (Dl) and dorsomedial (Dm) pallium have been identified as functional equivalents to the mammalian hippocampus and amygdala, respectively (Portavella et al., 2004; O’Connell and Hofmann, 2011; Broglio et al., 2015). The Dl and hippocampus play a role in relational memory of the environment and experiences, while the Dm and amygdala are involved in emotional learning and stress reactivity (Portavella et al., 2004; Vargas et al., 2009; O’Connell and Hofmann, 2011). Importantly, these proposed functional equivalences may in fact not be specific enough, since recent studies have suggested that the Dl and Dm are each composed of dorsal (Dld and Dmd) and ventral (Dlv and Dmv) neuronal subpopulations, each with distinct topology, connectivity patterns and, most likely, functionality (Broglio et al., 2015; Broglio, pers. comm.). The ventral part of the ventral telencephalon (Vv) has been suggested as the putative functional equivalent to the mammalian lateral septum (LS), which mediates social behavior and regulates goal-oriented behavior (O’Connell and Hofmann, 2011; Singewald et al., 2011). Together, the Dl, Dm, and Vv subregions (**Figure 1**) of the telencephalon are thus drivers of cognitive processes that are important for behavioral adaptation to novel environments.

To date, the few studies that compare the neurobiology of hatchery-reared fish to that of wild conspecifics have assessed neuroplasticity markers either at the level of the whole brain (Aubin-Horth et al., 2005) or large brain structures such as the hind- and mid-brain (Dunlap et al., 2011). While these studies indicated that the hatchery environment affects neuronal cell proliferation and gene expression patterns, it remains challenging to interpret how these neurobiological differences may be linked to behavior because, to our knowledge, no study has ever compared neuroplasticity markers between wild and hatchery-reared fish on the scale of specific neuronal populations. We sampled wild and hatchery-reared Atlantic salmon parr (juvenile freshwater fish) under basal and acute stress conditions in order to characterize their neurobiology in terms of *cfos* and *bdnf* transcript abundance in the Dld, Dlv, Dmd, Dmv, and Vv subregions of the telencephalon. We hypothesize that the rearing environment affects the expression of brain plasticity markers in these subregions, which are important for learning, memory and stress reactivity, and that this may, in part, explain the reported behavioral differences observed between wild and hatchery-reared salmonids. Here we present, for the first time, a detailed study that highlights differences in region-specific telencephalic gene expression between wild and hatchery-reared Atlantic salmon.

## Materials and Methods

### Ethics Statement

This experiment was performed under current Norwegian law for experimentation and procedures on live animals and was approved by the Norwegian Food Safety Authority (Mattilsynet) through FOTS application ID 10494.

### Animal Origin and Conditions

Hatchery-reared and wild Atlantic salmon parr were sampled at the Norwegian Institute for Nature Research (NINA) research station at Ims, Norway, and from the adjacent river Imsa on 12–13 September, 2016. The hatchery-reared fish were first generation offspring from wild parents from the river Imsa and thus of the same genetic origin as the sampled wild fish. These wild parents were captured from the river Imsa in November 2015, eggs and milt were harvested and cross-fertilized and the eggs hatched in late January 2016. Larvae started feeding mid-March in 4 m^3^ indoor tanks. Fish were transferred to a 50 m^3^ indoor concrete rearing tank at 5 months of age in June 2016. Approximately 300 of these parr were transferred to a 4-m^3^ holding tank 1 month prior to this experiment, where they were housed until sampling. Hatchery fish received flow-through natural water from the river Imsa, mixed with salt water to achieve a final salinity between 1and 2.5‰. Rearing salmon parr in very dilute saline water is a standard hatchery procedure due to its known health benefits, particularly in combatting freshwater fungi (Long et al., 1977). Juvenile fish were fed commercial feed (Nutra Parr, Skretting, Stavanger, Norway) throughout the day by an automatic feeder.

### Experimental Setup and Sampling

Wild and hatchery-reared salmon were collected at either basal or acute post-stress conditions (*n* = 7 per group; 28 fish in total). Both juvenile males and females were sampled and premature males were excluded: sex and premature maturation was verified by dissection after sampling. The sex ratios (M:F) for each group were as follows: hatchery basal: 5:2, hatchery stressed: 2:5, wild basal: 3:4, and wild stressed: 2:5. Wild parr were sampled on September 13 from the river Imsa, approximately 500 m upstream from the estuary (58.901385 and 5.957336), by electrofishing (Geomega type FA-4, Terik Technology, Levanger, Norway; 700 V). Electrofishing at this voltage does not kill the fish but merely stuns them for a few seconds, allowing enough time for capture. During electrofishing, we worked our way upstream to reduce the risk of catching fish that had previously been stunned and flushed downstream by the water current. Because wild fish were captured by electrofishing, we also subjected all hatchery-reared fish to a similar electric shock to reduce handling differences between treatments. To this end, prior to being sampled, hatchery-reared fish were individually collected by net from the 4 m^3^ holding tank and immediately transferred into a 150 l tank where they were stunned for 2 s with the same electrofishing equipment. All hatchery fish were sampled from the same tank on September 12. In order to minimize stress, repeated netting was avoided and the fish were processed immediately after netting. We verified that this capture procedure did not cause accumulative stress in the salmon in the holding tank from the observation that plasma cortisol levels of hatchery-reared fish did not increase throughout the sampling process, as the day progressed. In order to sample fish (both wild and hatchery) at basal conditions, individuals were anesthetized immediately after the electroshock in 0.75‰ (v/v) 2-phenoxyethanol (Sigma-Aldrich 77699), which rendered them unconscious within 30 s, at which point fish were quickly processed (see below). In order to sample fish post-stress, fish were subjected to a confinement stress, which is a commonly used paradigm which subjects fish to a standardized stressor (e.g., Pottinger et al., 1992). Individuals were subjected to a 30 min confinement stress by placing them in isolation in a 10 l bucket filled with 2 l of river water (bottom diameter: 200 mm and water depth: 65 mm). This confinement bucket was covered with a polystyrene lid and air was constantly supplied by a pump and a submersed aeration stone. The confinement bucket was rinsed thoroughly after every fish to remove any type of biological products that may have been excreted by previous fish. After confinement, stressed fish were anesthetized in the same way as described above for individuals collected at basal conditions and subsequently processed as described below. Fish at basal and stress conditions were sampled alternatingly to avoid time-of-day effects. After anesthetization, all fish were processed immediately: body mass and fork length were recorded and a blood sample was extracted from the caudal vein using cold heparinized syringes fitted with a 23G needle. Blood samples were kept on ice during sampling, followed by centrifugation for 5 min at 2,300 ×*g* to collect plasma, which was subsequently stored at −20°C for 2 days and then at −80°C until cortisol analysis. Fish were fixed by vascular perfusion with freshly made ice-cold 2% paraformaldehyde (PFA; Sigma-Aldrich P6148) in 0.1 M Sørensen’s phosphate buffer (PB; 28 mM NaH_2_PO_4_, 71 mM Na_2_HPO_4_, pH 7.2). Brains were then dissected out within 2 min and post-fixed overnight in 2% PFA in PB at 4°C. Brain tissue was washed three times for 20 min in Sørensen’s PB at room temperature and cryopreserved overnight in 25% sucrose (Sigma-Aldrich S9378) in Sørensen’s PB at 4°C. Tissues were then embedded in Tissue-Tek OCT compound (Sakura Finetek) in custom-made silicon molds, frozen on dry ice, wrapped in parafilm and stored at −80°C in 50 ml falcon tubes that contained 5 ml of frozen Milli-Q water to prevent dehydration.

### Cortisol Analysis

Plasma cortisol concentrations were determined by radioimmunoassay according to Gorissen et al. (2012). The primary antibody shows a 100% cross reactivity with cortisol, 0.9% with 11-deoxycortisol, 0.6% with cortiscosterone, and <0.01% with 11-deoxycorticosterone, progesterone, 17-hydroxyprogesterone, testosterone, and estradiol. All wells except the “non-specifics” received 100 μl cortisol antibody (cortisol antibody [SM210], monoclonal and IgG purified; Abcam Cat# ab1949, **RRID**:AB_302703); 1:2,000 and were incubated overnight at 4°C. The following day, the plates were washed three times with 200 μl/well wash buffer. Subsequently, non-specific sites were blocked by the addition of 100 μl blocking buffer to each well. Plates were covered and incubated for 1 h at 37°C. Subsequently, 10 μl of standard (4–2,048 pg cortisol/10 μl assay buffer or 10 μl of twice-diluted plasma was added to designated wells. Non-specifics and B_0_ wells received 10 μl assay buffer. After the addition of standards and samples, 333 Bq of ^3^H-hydrocortisone (PerkinElmer, United States, 1:10,000 in assay buffer) solution was added to each well. Plates were incubated for 4 h at room temperature, or stored overnight at 4°C. The plates were then washed three times with wash buffer. After the final wash step, all wells received 200 μl of “Optiphase hisafe-3 scintillation liquid” (PerkinElmer, United States) and were covered. Beta-emission was quantified by a 3 min count per well using a Microbeta Plus (Wallac/PerkinElmer, United States). Inter- and intra-assay variations were 12.5 and 3.5%, respectively.

### *In situ* Hybridization

*In situ* hybridization (ISH) for *cfos* and *bdnf* transcripts was performed on parallel sections for seven fish per treatment. For each fish, the telencephalon was sectioned transversely onto one Superfrost Ultra Plus slide (Menzel-Gläser) using a cryostat (Leica CM 3050) at −24°C. Sections were 14 μm thick and spaced 90 μm apart. Slides were dried at 60°C for 10 min and subsequently stored at −80°C until further analysis. The ISH digoxigenin-labeled probes were made according to Vindas et al. (2017) and were 906 and 485 nucleotides long for *cfos* and *bdnf*, respectively. Forward ACTCCGCTTTCAACACCGAC and reverse TGTAGAGAGGCTCCCAGTCC and forward TCACAGACACGTTTGAGCAGGTGA and reverse ATGCCTCTTGTCTATTCCACGGCA primers were used for *cfos* and *bdnf* probes, respectively. The ISH protocol was conducted according to Ebbesson et al. (2011). Slides were mounted in 70% glycerol in 10 mM Tris-HCl (pH 7.5), 1 mM EDTA, and 150 mM NaCl. For both *cfos* and *bdnf*, all 28 slides were stained simultaneously in the same Coplin staining jars in random positions to avoid differences in coloration due to handling effects. Alignment of the probe sequences (**Supplementary Presentation S1**) in BLAST revealed a 100% similarity with several predicted *Salmo salar* *bdnf* transcripts (XM014175921.1 and others) and 99% similarity with the predicted *Salmo salar* *cfos* transcript (XM014206157.1). Both sense and antisense probes were tested to confirm specific labeling of target genes (**Supplementary Figure S1**).

### Quantification of Labeled Cells

After ISH, slides were photographed using an Axio Scan.Z1 slide scanner (Zeiss) at 20× magnification. Labeled *cfos* and *bdnf* cells were quantified using the Fiji platform (Schindelin et al., 2012; **RRID**:SCR_002285) in ImageJ2 (Rueden et al., 2017; **RRID**:SCR003070). Brain regions were identified using several salmonid stereotaxic atlases (Navas et al., 1995; Carruth et al., 2000; Northcutt, 2006) and transcript-positive cells were counted in the Dl (both the dorsal and ventral subregions; Dld and Dlv, respectively) and Dm (both the dorsal and ventral subregions; Dmd and Dmv, respectively) pallium, as well as in the Vv (see **Figure 1** for an overview of the subregions). An Image J macro script was developed to semi-automate quantification of labeled cells (**Supplementary Presentation S2**). In short, images were converted into grayscale (8 bit), the area of interest was manually selected and the black and white threshold was adjusted within the range of 145 and 190 to match the labeled cells in the original image. Then, all labeled cells that measured between 15 and 500 pixels were counted using the “Analyze Particles” command. Example images of the semi-automated quantification method are provided in **Supplementary Figure S2**. For each section, the total number of transcript-labeled cells was counted in both the entire Dl and Dm, as well as within their respective dorsal and ventral subregions (Dld and Dlv, Dmd and Dmv), to elucidate subregion-specific expression patterns and to allow for comparisons with previous studies (e.g., Vindas et al., 2017). The number of labeled cells was quantified as described by Vindas et al. (2018) and Moltesen et al. (2016). In short, the number of transcript-expressing cells was counted within each subregion for both lobes in each section (in which interest areas were found). Labeled cells were counted in 9.0 ± 1.4 (mean ± SD) telencephalon sections per fish and because the number of brain sections differed per fish, we corrected for the number of counted sections by calculating the average number of labeled cells per section for each subregion. These average numbers of labeled cells per section in each area were used in the statistical analysis. Samples were quantified in random order and the experimenter did not know the identity of the samples at time of quantification.

### Statistical Analyses

Two-way analysis of variance (ANOVA) was used to compare fork length, body mass, plasma cortisol levels and ISH cell counts, with origin (wild vs. hatchery) and treatment (basal vs. stress) as independent variables. The fish telencephalon consists of two lobes (**Figure 1**). To test whether lateralization preferences occurred (i.e., different neural responses in the left vs. right telencephalic lobe), the labeled cells were quantified in each lobe separately and for each area of interest, we tested if the left and right cell counts were statistically different from each other (Spearman’s ρ). Because we did not find any significant differences in labeled cell numbers between the two lobes, the cell quantifications of the two lobes were pooled together for further statistical analysis and the absolute number of transcript-expressing cells were compared between treatments. Models were assessed by their capacity to explain the variability, and the interaction effects between treatment and conditions were accepted or rejected according to total model “lack of fit” probabilities. Upon inspection of the diagnostic residual plots, all ISH cell counts and cortisol values were ^10^log transformed before statistical analysis. Tukey–Kramer honestly significant difference (HSD) *post-hoc* tests were conducted for brain areas that showed both a significant origin and treatment effect or a significant interaction effect, in order to elucidate differences between groups. Individual data points are shown, as well as the mean ± standard error of the mean (SEM).

## Results

### Body Size and Plasma Control

**FIGURE 2:**
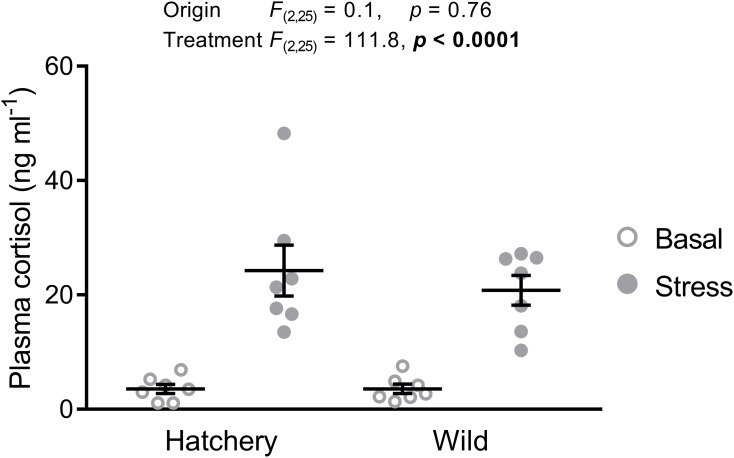
Acute stress elevates plasma cortisol levels. Effect of origin (hatchery vs. wild) and treatment (basal vs. stress) on mean ± SEM plasma cortisol levels of Atlantic salmon parr. Two-way analysis of variance (ANOVA) statistics are displayed in the figure, *n* = 7 per treatment.

As expected, wild fish were significantly smaller (fork length: 81 ± 2 mm vs. 112 ± 1 mm; *p* < 0.0001) and weighed less (6.7 ± 0.6 g vs. 16.8 ± 0.8 g; *p* < 0.0001) than hatchery-reared fish. Fork length (*p* = 0.68) and body mass (*p* = 0.94) did not significantly differ between stressed and basal fish. Basal plasma cortisol levels were approximately 3.6 ng ml^−1^ for both hatchery and wild parr (**Figure 2**). The 30-min confinement stress significantly elevated plasma cortisol levels to 24.3 ± 4.5 ng ml^−1^ and 20.8 ± 2.6 ng ml^−1^ in hatchery and wild fish, respectively (treatment effect: *p* < 0.0001). No origin or interaction effect was found (*p* = 0.76 and *p* = 0.61, respectively).

### Expression of *cfos* and *bdnf*

#### The Dorsolateral Pallium and Its Subregions

**FIGURE 3:**
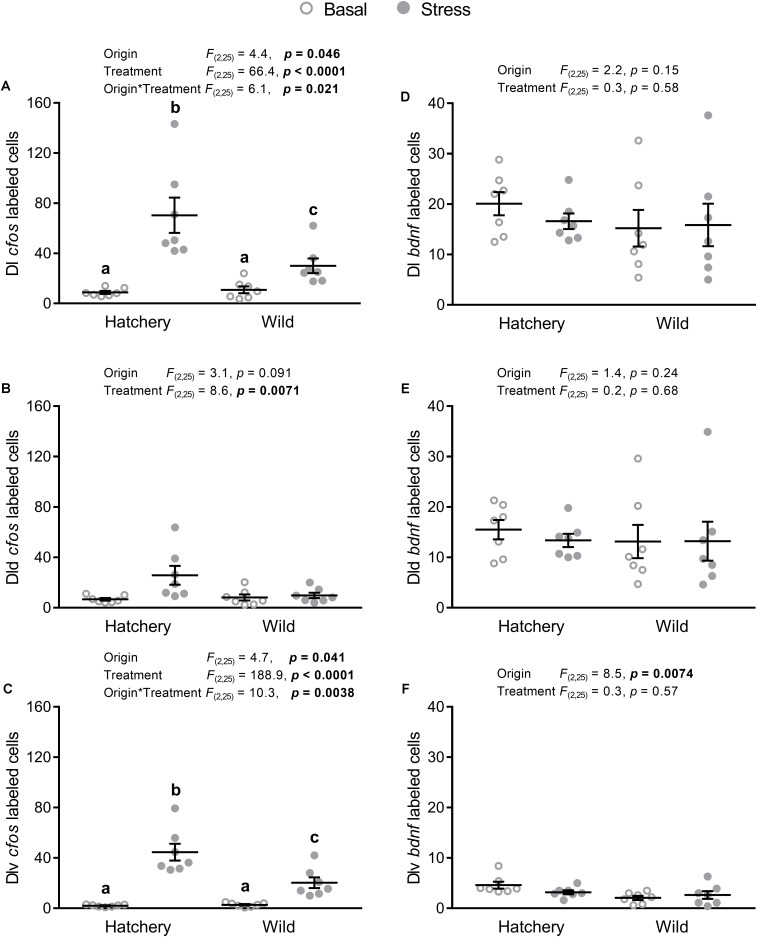
*In situ* hybridization labeled *cfos* and *bdnf* cells in the Dl, Dld, and Dlv. Effect of origin (hatchery vs. wild) and treatment (basal vs. stress) on mean ± SEM expression of *cfos* **(A–C)** and brain-derived neurotrophic factor (*bdnf*; **D–F**) in the entire (dorsal + ventral) dorsolateral pallium (Dl; **A,D**), as well as the dorsal only (Dld; **B,E**) and the ventral only (Dlv; **C,F**) subregions. Two-way analysis of variance (ANOVA) statistics are displayed in each panel, *n* = 7 per treatment. Groups that do not share a similar lowercase letter are significantly different from one another (Tukey–Kramer HSD *post-hoc* test).

*In situ* hybridization analysis of *cfos* in the Dl as a whole (**Figure 3A**) revealed a significant origin (*p* = 0.046), treatment (*p* < 0.0001), and interaction effect (*p* = 0.021), with a significantly higher absolute number of *cfos*-labeled cells post-stress in both hatchery (*p* < 0.0001) and wild (*p* = 0.0027) fish, compared to basal conditions. In addition, post-stress hatchery fish had a higher number of *cfos*-labeled cells in the Dl compared to post-stress wild fish (*p* = 0.017). In the Dld (**Figure 3B**), a treatment effect showed overall more *cfos*-labeled cells in response to stress compared to basal conditions (*p* = 0.0071). No effect of origin was found (*p* = 0.091). The Dlv (**Figure 3C**) showed a similar pattern as the whole Dl, with a significant origin (*p* = 0.041), treatment (*p* < 0.0001), and interaction effect (*p* = 0.0038). *Post-hoc* analysis revealed higher *cfos* expression in response to stress for both hatchery (*p* < 0.0001) and wild (*p* < 0.0001) individuals, compared to values at basal conditions. Furthermore, post-stress hatchery fish had a higher number of *cfos*-positive cells in the Dlv compared to post-stress wild individuals (*p* = 0.0045).

*In situ* hybridization analysis of *bdnf* in the Dl as a whole (**Figure 3D**) and in the Dld (**Figure 3E**) revealed no significant origin or treatment effects. Meanwhile, there was a significant origin effect (*p* = 0.0074) in the Dlv (**Figure 3F**), with hatchery fish showing overall higher numbers of *bdnf*-labeled cells compared to wild fish.

#### The Dorsomedial Pallium and Its Subregions

**FIGURE 4:**
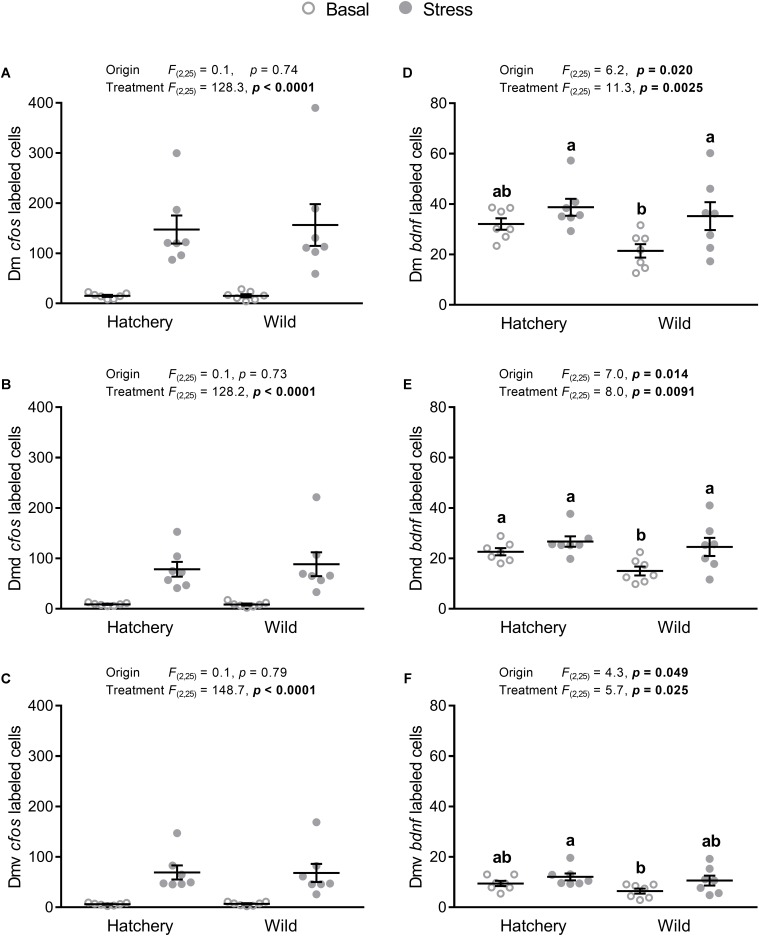
*In situ* hybridization labeled *cfos* and *bdnf* cells in the Dm, Dmd, and Dmv. Effect of origin (hatchery vs. wild) and treatment (basal vs. stress) on mean ± SEM expression of *cfos* **(A–C)** and brain-derived neurotrophic factor (*bdnf*; **D–F**) in the entire (dorsal + ventral) dorsomedial pallium (Dm; **A,D**), as well as the dorsal only (Dmd; **B,E**) and the ventral only (Dmv; **C,F**) subregions. Two-way analysis of variance (ANOVA) statistics are displayed in each panel, *n* = 7 per treatment. Groups that do not share a similar lowercase letter are significantly different from one another (Tukey–Kramer HSD *post-hoc* test).

There was a significant treatment effect on *cfos* expression in the Dm, Dmd, and Dmv (*p* < 0.0001 in all areas; **Figures 4A–C**), showing a higher *cfos* transcript abundance in stressed fish. No origin effects were found for the Dm, Dmd, or the Dlv.

Expression of *bdnf* showed a significant origin (*p* = 0.020, *p* = 0.014, and *p* = 0.049) and treatment (*p* = 0.0025, *p* = 0.0091, and *p* = 0.025) effect for the Dm (**Figure 4D**), Dmd (**Figure 4E**), and Dmv (**Figure 4F**) respectively, where hatchery fish showed an overall higher *bdnf* abundance compared to wild individuals and post-stress *bdnf* expression was higher compared to values at basal levels. Tukey–Kramer HSD *post-hoc* tests revealed that in the Dm, the wild group at basal conditions showed significantly lower *bdnf* expression compared to the wild stressed (*p* = 0.0129) and hatchery stressed (*p* = 0.0017) groups, but not to the hatchery basal group (*p* = 0.053). In the Dmd, the wild basal group had a significantly lower number of *bdnf*-labeled cells compared to all three other groups (*p* = 0.038, *p* = 0.0032, *p* = 0.027 for wild basal vs. hatchery basal, hatchery stressed, and wild stressed, respectively). In the Dmv, the wild basal group showed a significantly lower number of *bdnf*-labeled cells than the hatchery stressed group (*p* = 0.0435) and no other significant differences were found between groups.

#### The Ventral Part of the Ventral Telencephalon

**FIGURE 5:**
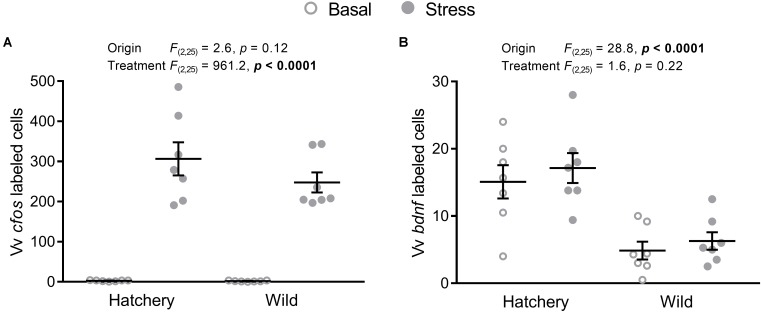
*In situ* hybridization labeled *cfos* and *bdnf* cells in the Vv. Effect of origin (hatchery vs. wild) and treatment (basal vs. stress) on mean ± SEM expression of *cfos* **(A)** and brain-derived neurotrophic factor (*bdnf*; **B**) in the ventral part of the ventral telencephalon (Vv). Two-way analysis of variance (ANOVA) statistics are displayed in each panel, *n* = 7 per treatment.

In the Vv, *cfos* (**Figure 5A**) expression was significantly elevated overall in response to stress (*p* < 0.0001), while no significant effects of origin were observed (*p* = 0.12).

The number of *bdnf*-labeled cells (**Figure 5B**) in the Vv was overall significantly higher in hatchery fish (*p* < 0.0001) compared to wild individuals and no treatment effects were found (*p* = 0.22).

**FIGURE 6:**
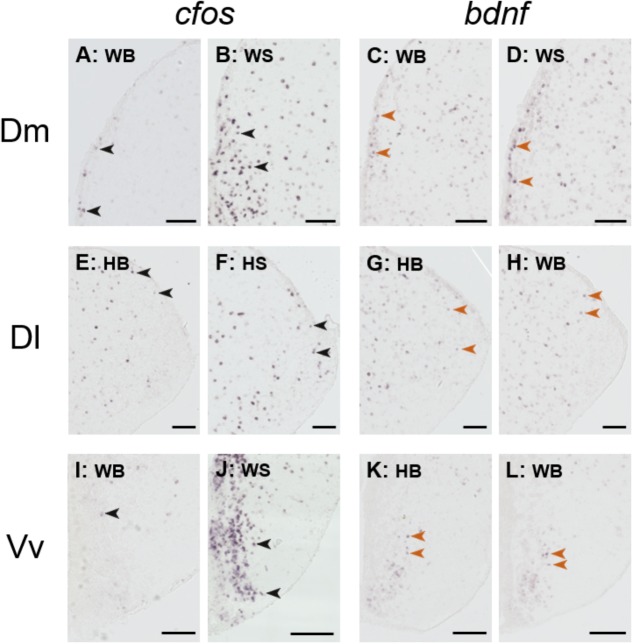
Representative example of *in situ* hybridization of *cfos* and *bdnf;* images used for the quantification analysis. Representative pictures of the expression of *cfos* **(A,B,E,F,I,J)** and brain-derived neurotrophic factor (*bdnf*; **C,D,G,H,K,L**) transcripts (*purple* cells) in the dorsomedial pallium (Dm; **A–D**), dorsolateral pallium (Dl; **E–H**), and ventral part of the ventral telencephalon (Vv; **I–L**) of wild and hatchery-reared Atlantic salmon parr under basal or after acute stress conditions. WB, wild basal; WS, wild stress; HB, hatchery-reared basal; HS, hatchery-reared stress. Arrows indicate transcript-labeled cells and all scale bars measure 100 μm.

**Figure 6** depicts representative examples of ISH images that were used for the quantification analysis.

## Discussion

We found distinct differences in region-specific expression of *cfos* and *bdnf* in the telencephalon of hatchery-reared and wild Atlantic salmon parr under basal and acute stress conditions. While the stressor resulted in increased *cfos* abundance in all fish, hatchery-reared individuals showed a significantly stronger increase in *cfos*-positive cells than wild fish in the ventral part of the dorsolateral pallium (Dlv). Transcript abundance of *bdnf* increased in response to acute stress in the dorsal part of the dorsomedial pallium (Dmd) of wild fish, but not in that of hatchery-reared individuals. Thus, our findings demonstrate that neuronal activity and neural plasticity in Atlantic salmon is dependent on both origin (i.e., wild or hatchery-reared) and treatment conditions (i.e., basal or post-acute stress) and that these processes differ in a region-specific manner. To our knowledge, we are the first to map neuronal differences between wild and hatchery-reared fish within telencephalic subregions and our results provide novel insights into the neurological foundation that could underlie the differences in behavior, and stocking success, between wild and hatchery-reared fish.

Plasma cortisol levels in stressed parr increased approximately sixfold compared to controls and the range of average plasma cortisol concentrations found in this study (3–25 ng ml^−1^) was within the range of those previously reported for non-migratory Atlantic salmon parr (Carey and McCormick, 1998; McCormick et al., 2000; Madaro et al., 2015, 2016). Because hatchery-reared fish are subject to human disturbance more frequently than wild fish, we had hypothesized that hatchery fish would habituate more easily to stress and therefore show a mitigated cortisol response to a stressor. However, hatchery-reared and wild salmon showed no differences in plasma cortisol concentrations at either basal or post-stress conditions. Similar plasma cortisol levels for wild and hatchery-reared salmonids at 30 min post-stress have previously been reported for rainbow trout (*Oncorhynchus mykiss*) (Woodward and Strange, 1987), suggesting that the magnitude of the immediate cortisol response to acute stress is not affected by hatchery rearing. However, several studies report higher plasma cortisol concentrations in wild salmonids [rainbow trout, Coho salmon (*O. kisutch*), and Chinook salmon (*O. tshawytscha*)] or salmon reared in semi-natural rearing environments (*O. tshawytscha*) compared to hatchery-reared conspecifics at 1–12 h post-stress, suggesting that recovery of cortisol to baseline levels is slower in wild individuals (Woodward and Strange, 1987; Salonius and Iwama, 1993; Madison et al., 2015). These findings thus suggest that, despite the fact that we observed a similar cortisol response in wild and hatchery-reared fish at 30 min post-stress, it is possible that hatchery individuals recover from the acute stress more quickly, which would indicate that the hatchery environment alters the long-term endocrine stress response. Further studies should confirm this hypothesis by assessing cortisol peak levels, as well as recovery duration, in wild vs. hatchery Atlantic salmon populations, to determine the effect of hatchery rearing on stress coping.

The IEG *cfos* is a robust marker for recent neural activity (Okuno, 2011). Within specific neuronal populations, the *cfos* gene is relatively little expressed at basal levels but when neurons are stimulated, *cfos* expression is rapidly increased with mRNA levels typically peaking between 15 and 30 min post activation (Hoffman et al., 1993; Pavlidis et al., 2015). Acute stress can increase neuronal *cfos* expression in a variety of animals, including rats (Cullinan et al., 1995; Rosen et al., 1998), zebrafish (*Danio rerio*; Pavlidis et al., 2015), gilthead seabream (*Sparus aurata*; Vindas et al., 2018) and Atlantic salmon (Vindas et al., 2017). Our findings corroborate that acute stress increases *cfos* expression in the Dl, Dm, and Vv of teleost fish (Vindas et al., 2017, 2018). Increased *cfos* expression post-acute stress has also been reported in mammalian limbic areas, including in brain regions which are functionally equivalent to the fish Dl, Dm, and Vv (Cullinan et al., 1995). Recent studies have suggested that the Dlv, not the Dld, bears most resemblance to the mammalian hippocampus (Broglio et al., 2015). Therefore, we quantified *cfos* expression separately in the dorsal and ventral subregions of the Dl and our observation that *cfos* shows a different expression pattern in the Dlv (treatment, origin, and interaction effect) compared to the Dld (treatment effect only) supports the hypothesis that the Dld and Dlv are associated with the regulation of different processes. Research on mice has shown that a fear conditioning stimulus increases CFOS expression in hippocampal cells, and when these same cells are reactivated through optogenetic stimulation the mice display freezing behavior, demonstrating that hippocampal CFOS expression is involved with neural activity associated with fear memory storage and retrieval (Liu et al., 2012). We observed that reared fish showed a greater increase of *cfos* expression in the Dlv in response to acute stress compared to wild individuals. Reared salmonids often show reduced antipredator performance compared to wild conspecifics (Huntingford, 2004). As the hippocampus plays an important role in mammalian fear memory and retrieval, and we observe different responsiveness of the Dlv to confinement stress between hatchery-reared and wild fish, it would be interesting to assess whether wild and reared fish would also display differences in neural activation in the Dlv in a fear-conditioning test, and how this may relate to important behavioral paradigms such as antipredator behavior. Finally, the Dlv also plays an important role in spatial memory. That is, lesions in the Dlv result in place-memory deficits in goldfish (*Carassius auratus*; Rodrìguez et al., 2002; Broglio et al., 2010) in a similar way that lesions of the hippocampus reduce the navigating capacity of mammals (Hampton et al., 2004). Therefore, it is likely that the Dlv is important for navigating between natural foraging grounds. In this context, it would be interesting to examine whether the difference in post-stress activation of the Dlv that we found between wild and hatchery-reared fish is associated with their ability to navigate, learn and retrieve memories on foraging patches and prey abundance, particularly in a risky environment (e.g., under threat from predators).

Brain-derived neurotrophic factor is a protein from the neurotrophin family that promotes synaptic plasticity, long-term potentiation, neurogenesis, and cell survival (Mattson et al., 2004; Pang et al., 2004; Gray et al., 2013). In the whole fish brain, *bdnf* mRNA is generally upregulated in response to acute stress (Pavlidis et al., 2015) and downregulated after chronic stress (Tognoli et al., 2010). Mammalian studies show that changes in *BDNF* expression in response to external stimuli are region-specific within the central nervous system. For example, while both chronic and acute stress result in a significant elevation of BDNF protein abundance in the mammalian amygdala, stress can decrease BDNF levels in the hippocampus (Gray et al., 2013). While mammalian studies almost exclusively study region-specific expression patterns of *BDNF*, studies on teleosts often target *bdnf* expression in the whole brain or macro-brain regions such as the whole telencephalon or whole cerebellum (Tognoli et al., 2010; Johansen et al., 2012; Pavlidis et al., 2015). In these teleostean studies, acute stress increased *bdnf* expression in the whole brain of zebrafish (Pavlidis et al., 2015), while it did not alter *bdnf* transcript abundance in the whole telencephalon of rainbow trout (Johansen et al., 2012) nor in the whole brain of European sea bass, *Dicentrarchus labrax* (Tognoli et al., 2010). Interestingly, while we did not find a change in *bdnf* expression in the Dl and Vv in response to stress in any of our study groups, consistent with the findings of Johansen et al. (2012) and Tognoli et al. (2010), we did find significantly more *bdnf*-labeled cells in the Dm of wild stressed individuals, supporting the findings by Pavlidis et al. (2015). Furthermore, the increase in post-stress *bdnf* expression in the Dm is in agreement with the finding that BDNF abundance is increased post-stress in the mammalian amygdala (Gray et al., 2013). This result is interesting to study further, particularly because this emotional/stress reactivity center may play a significant role in predator recognition and negative stimuli avoidance conditioning (Portavella et al., 2004). Additionally, this finding further demonstrates that in salmon, as has been shown earlier (Vindas et al., 2017), targeting neuronal subregions can reveal expression patterns that escape detection when studying whole brains or whole macro-brain areas such as the entire telencephalon. Interestingly, as with *cfos* expression in the Dl, the dorsal (Dmd) and ventral (Dmv) neural populations of the Dm in wild fish showed different *bdnf* expression profiles. That is, while acute stress increased *bdnf* expression in the Dmd, it did not in the Dmv. This observation raises the possibility that, similar to what has been proposed for the neural subpopulations of the Dl, the dorsal and ventral subregions of the Dm have different functionalities also, as suggested by preliminary work by Broglio (pers. comm.). Finally, even though hatchery-reared fish did not show any increase in *bdnf* in response to stress, we observed that this group showed an overall higher expression of *bdnf* in the Dm, the Vv, and the Dlv, compared to wild individuals, with the highest number of *bdnf*-labeled cells present in the Dm, which plays an important role in learning under fear and stress (Portavella et al., 2004; Vargas et al., 2009; O’Connell and Hofmann, 2011). Under hatchery conditions, fish regularly experience disturbances (e.g., tank cleaning, grading, vaccination, transport, etc.) at unpredictable intervals. It is conceivable that these stressors periodically trigger *bdnf* transcription in the Dm of hatchery fish, effectively elevating basal *bdnf* expression levels in this subregion. The Dm shares reciprocal neuronal connections with the Vv, which in turn connects to the Dlv (Folgueira et al., 2004a,b; Northcutt, 2006). Notably, mammalian research has demonstrated that the amygdala and hippocampus play an important regulatory role in the hypothalamo-pituitary-adrenal (HPA) axis (McEwen, 2003), and that the LS and amygdala are both part of a circuit involved with stress-induced anxiety behavior (Anthony et al., 2014). Extrapolating our results to these mammalian findings, we propose that the frequent disturbances associated with life in an anthropogenic environment (i.e., hatchery rearing) increases *bdnf* expression at basal conditions in the Dm, which in turn promotes *bdnf* expression in the Dl and Vv through neural circuits that are involved in the stress axis. Together with the fact that hatchery-reared fish did not show an increase in *bdnf* in response to stress, and the fact that increased BDNF levels are linked to a higher learning performance in mammals (Vaynman et al., 2004), our results may show a potential reduced capacity for learning performance in hatchery-reared fish under acute stressful conditions, which would affect their post-release survival, particularly in risky environments (e.g., under predator pressure). Future studies should examine the learning performance and *bdnf* transcription of individuals under stress and non-stress conditions, to elucidate whether our observation that there are different *bdnf* expression patterns between wild and hatchery fish in the Dm affects their emotional learning response.

To our knowledge, no other studies had ever compared neurobiological markers between wild and hatchery-reared salmonids using a detailed region-specific approach. For this reason, we selected ISH as our methodology, since it allows for the visualization of transcript abundance of target genes in the entire brain, providing a clear overview of which neural subpopulations can be of interest. The disadvantage of using ISH to quantify expression of neurobiological markers is that the quantification process in our analysis is binary: cells are either classified as expressing or non-expressing, while the relative transcript abundance within the cells is not considered. In order to map gene expression patterns in a more quantitative manner, future studies should perform microdissections of the relevant forebrain subregions and subsequently quantify the transcript abundance by quantitative polymerase chain reaction (qPCR), as we have previously done when studying the neurobiological component of coping styles in Atlantic salmon (Vindas et al., 2017). By using ISH in the current study, we were able to compare region-specific expression of neurobiological markers between wild and hatchery-reared fish on the most detailed scale to date. The studied neuronal subpopulations are involved in learning processes and stress reactivity and thus provide an important insight in how neural plasticity may drive behavioral differences between wild and hatchery-reared fish.

## Conclusion

We demonstrate that the rearing environment is an important driver of neuronal wiring in the telencephalon of Atlantic salmon parr. We show novel data on expression of neuroplasticity markers within specific neuronal subregions in wild and hatchery-reared fish and this approach has unveiled stress-related expression patterns that have previously escaped detection (i.e., when studying larger brain areas; Johansen et al., 2012; Pavlidis et al., 2015). The specific brain areas mapped in the current study are associated with cognitive processing capacity (specifically stress reactivity, associative learning, and emotional learning) and may therefore play an important role in the behavioral differences that are observed between wild and hatchery-reared teleosts. A better understanding of how the rearing environment affects the neurological and behavioral plasticity of captive animals will help with the future design of innovative hatchery technologies that produce well-adapted salmonids that can thrive after stocking. In addition, these results provide further insight into mechanisms of the central nervous system associated with behavioral processing and coping in vertebrates and provides focal areas which should be studied further to elucidate how animals react to, and interact with, their environment.

## Data Availability

All relevant data are within the paper or published as Supplementary Material, see **Supplementary Datasheets S1**, **S2**.

## Author Contributions

DM, KvK, IM, and MV conceived and designed the study. DM and KvK collected the samples. DM, MG, and MV performed laboratory analyses. DM and MV conducted statistical analysis. DM and MV wrote the first draft of the manuscript. All authors contributed significantly to manuscript revision and approved the final version.

## Conflict of Interest Statement

The authors declare that the research was conducted in the absence of any commercial or financial relationships that could be construed as a potential conflict of interest.

## Supplementary Material

FIGURE S1 Specificity of the riboprobes was verified by performing the *in situ* hybridization protocol using both sense and antisense probes on a subset of brain samples. Depicted are the sense **(A)** and antisense **(B)** probes for *bdnf* and the sense **(C)** and antisense **(D)** probes for *cfos*.

FIGURE S2 Illustration of several steps of the semi-automated quantification method of transcript-positive cells. The area of interest was selected using the selection tool **(A)**, the black and white threshold was set to match the original image as closely as possible **(B)**, and all cells that measures between 15 and 500 pixels were counted by the ImageJ software **(C)**. *Orange arrows* indicate the location of a cell which is labeled too weakly to be quantified by the software after adjusting the black and white threshold **(B)**.

PRESENTATION S1 Sequences of the riboprobes of *bdnf* and *cfos* used for *in situ* hybridization.

PRESENTATION S2 The macro script was used for quantification of labeled cells using the Fiji platform in ImageJ2.

DATA SHEET S1 *bdnf* and *cfos*.

DATA SHEET S2 Average number of *cfos*-expressing cells per section.

## References

[B1] Anthony T. E. Dee N. Bernard A. Lerchner W. Heintz N. Anderson D. J. (2014). Control of stress-induced persistent anxiety by an extra-amygdala septohypothalamic circuit. *Cell* 156 522–536. 10.1016/j.cell.2013.12.040 24485458 PMC3982923

[B2] Araki H. Schmid C. (2010). Is hatchery stocking a help or harm? *Aquaculture* 308 S2–S11. 10.1016/j.aquaculture.2010.05.036

[B3] Aubin-Horth N. Letcher B. H. Hofmann H. A. (2005). Interaction of rearing environment and reproductive tactic on gene expression profiles in Atlantic salmon. *J. Hered.* 96 261–278. 10.1093/jhered/esi030 15653555

[B4] Broglio C. Martín-Monzón I. Ocaña F. Gómez A. Durán E. Salas C. (2015). Hippocampal pallium and map-like memories through vertebrate evolution. *J. Behav. Brain. Sci.* 5 109–120. 10.4236/jbbs.2015.53011

[B5] Broglio C. Rodríguez F. Gómez A. Arias J. L. Salas C. (2010). Selective involvement of the goldfish lateral pallium in spatial memory. *Behav. Brain Res.* 210 191–201. 10.1016/j.bbr.2010.02.031 20178818

[B6] Brown G. E. Ferrari M. C. O. Chivers D. P. (2013). Adaptive forgetting: why predator recognition training might not enhance poststocking survival. *Fisheries* 38 16–25. 10.1080/03632415.2013.750133

[B7] Bshary R. Brown C. (2014). Fish cognition. *Curr. Biol.* 24 R947–R950. 10.1016/j.cub.2014.08.043 25291632

[B8] Carey J. B. McCormick S. D. (1998). Atlantic salmon smolts are more responsive to an acute handling and confinement stress than parr. *Aquaculture* 168 237–253. 10.1016/S0044-8486(98)00352-4

[B9] Carruth L. L. Jones R. E. Norris D. O. (2000). Cell density and intracellular translocation of glucocorticoid receptor-immunoreactive neurons in the kokanee salmon (*Oncorhynchus nerka* kennerlyi) brain, with an emphasis on the olfactory system. *Gen. Comp. Endocrinol.* 117 66–76. 10.1006/gcen.1999.7391 10620424

[B10] Cullinan W. E. Herman J. P. Battaglia D. F. Akil H. Watson S. J. (1995). Pattern and time course of immediate early gene expression in rat brain following acute stress. *Neuroscience* 64 477–505. 10.1016/0306-4522(94)00355-9 7700534

[B11] Dunlap K. D. Silva A. C. Chung M. (2011). Environmental complexity, seasonality and brain cell proliferation in a weakly electric fish, *Brachyhypopomus gauderio*. *J. Exp. Biol.* 214 794–805. 10.1242/jeb.051037 21307066 PMC3036548

[B12] Ebbesson L. O. Braithwaite V. A. (2012). Environmental effects on fish neural plasticity and cognition. *J. Fish Biol.* 81 2151–2174. 10.1111/j.1095-8649.2012.03486.x 23252732

[B13] Ebbesson L. O. E. Nilsen T. O. Helvik J. V. Tronci V. Stefansson S. O. (2011). Corticotropin-releasing factor neurogenesis during midlife development in salmon: genetic, environmental and thyroid hormone regulation. *J. Neuroendocrinol.* 23 733–741. 10.1111/j.1365-2826.2011.02164.x 21592238

[B14] Folgueira M. Anadón R. Yáñez J. (2004a). An experimental study of the connections of the telencephalon in the rainbow trout (*Oncorhynchus mykiss*). I: olfactory bulb and ventral area. *J. Comp. Neurol.* 480 180–203. 10.1002/cne.20340 15514934

[B15] Folgueira M. Anadón R. Yáñez J. (2004b). Experimental study of the connections of the telencephalon in the rainbow trout (*Oncorhynchus mykiss*). II: dorsal area and preoptic region. *J. Comp. Neurol.* 480 204–233. 10.1002/cne.20341 15514931

[B16] Gorissen M. Bernier N. J. Manuel R. de Gelder S. Metz J. R. Huising M. O. (2012). Recombinant human leptin attenuates stress axis activity in common carp (*Cyprinus carpio* L.). *Gen. Comp. Endocrinol.* 178 75–81. 10.1016/j.ygcen.2012.04.004 22543190

[B17] Gray J. D. Milner T. A. McEwen B. S. (2013). Dynamic plasticity: the role of glucocorticoids, brain-derived neurotrophic factor and other trophic factors. *Neuroscience* 239 214–227. 10.1016/j.neuroscience.2012.08.034 22922121 PMC3743657

[B18] Hampton R. R. Hampstead B. M. Murray E. A. (2004). Selective hippocampal damage in rhesus monkeys impairs spatial memory in an open-field test. *Hippocampus* 14 808–818. 10.1002/hipo.10217 15382251

[B19] Hoffman G. E. Smith M. S. Verbalis J. G. (1993). c-Fos and related immediate early gene products as markers of activity in neuroendocrine systems. *Front. Neuroendocrinol.* 14 173–213. 10.1006/frne.1993.1006 8349003

[B20] Huntingford F. A. (2004). Implication of domestication and rearing conditions for the behaviour of cultivated fishes. *J. Fish Biol.* 65 122–142. 10.1111/j.1095-8649.2004.00562.x

[B21] Johansen I. B. Sorensen C. Sandvik G. K. Nilsson G. E. Hoglund E. Bakken M. (2012). Neural plasticity is affected by stress and heritable variation in stress coping style. *Comp. Biochem. Physiol. Part D Genomics Proteomics* 7 161–171. 10.1016/j.cbd.2012.01.002 22285148

[B22] Johnson J. H. McKeon J. E. Dropkin D. S. (1996). Comparative diets of hatchery and wild Atlantic salmon smolts in the Merrimack river. *N. Am. J. Fish. Manage.* 16 440–444.

[B23] Johnsson J. I. Brockmark S. Näslund J. (2014). Environmental effects on behavioural development consequences for fitness of captive-reared fishes in the wild. *J. Fish Biol.* 85 1946–1971. 10.1111/jfb.12547 25469953

[B24] Jonsson B. Jonsson N. (2009). “Restoration and enhancement of salmonid populations and habitats with special reference to Atlantic salmon,” in *Challenges for Diadromous Fishes in a Dynamic Global Environment*, eds Haro A. J. Avery T. S. Beal K. L. (Bethesda, ML: American Fisheries Society Symposium), 497–535.

[B25] Kalueff A. V. Stewart A. M. Kyzar E. J. Cachat J. Gebhardt M. Landsman S. (2012). Time to recognize zebrafish ‘affective’ behavior. *Behaviour* 149 1019–1036. 10.1163/1568539X-00003030

[B26] Liu X. Ramirez S. Pang P. T. Puryear C. B. Govindarajan A. Deisseroth K. (2012). Optogenetic stimulation of a hippocampal engram activates fear memory recall. *Nature* 484 381–385. 10.1038/nature11028 22441246 PMC3331914

[B27] Long C. W. McComas J. R. Monk B. H. (1977). Use of salt (NaCl) water to reduce mortality of Chinook salmon smolts, Oncorhynchus Tshawytscha, during handling and hauling. *Mar. Fish. Rev.* 39 6–9.

[B28] Madaro A. Olsen R. E. Kristiansen T. S. Ebbesson L. O. E. Flik G. Gorissen M. (2016). A comparative study of the response to repeated chasing stress in Atlantic salmon (Salmo salar L.) parr and post-smolts. *Comp. Biochem. Physiol. A Mol. Integr. Physiol.* 192 7–16. 10.1016/j.cbpa.2015.11.005 26549876

[B29] Madaro A. Olsen R. E. Kristiansen T. S. Ebbesson L. O. E. Nilsen T. O. Flik G. (2015). Stress in Atlantic salmon: response to unpredictable chronic stress. *J. Exp. Biol.* 218 2538–2550. 10.1242/jeb.120535 26056242

[B30] Madison B. N. Heath J. W. Heath D. D. Bernier N. J. (2015). Effects of early rearing environment and breeding strategy on social interactions and the hormonal response to stressors in juvenile Chinook salmon. *Can. J. Fish. Aquat. Sci.* 72 673–683. 10.1139/cjfas-2014-0409

[B31] Mattson M. P. Maudsley S. Martin B. (2004). BDNF and 5-HT: a dynamic duo in age-related neuronal plasticity and neurodegenerative disorders. *Trends Neurosci.* 27 589–594. 10.1016/j.tins.2004.08.001 15374669

[B32] Maynard D. J. Trial J. G. (2013). The use of hatchery technology for the conservation of Pacific and Atlantic salmon. *Rev. Fish Biol. Fish.* 24 803–817. 10.1007/s11160-013-9341-7

[B33] McCormick S. D. Moriyama S. Björnsson B. T. (2000). Low temperature limits photoperiod control of smolting in Atlantic salmon through endocrine mechanisms. *Am. J. Physiol. Regul. Integr. Comp. Physiol.* 278 R1352–R1361. 10.1152/ajpregu.2000.278.5.R1352 10801307

[B34] McEwen B. S. (2003). Early life influences on life-long patterns of behavior and health. *Ment. Retard. Dev. Disabil. Res. Rev.* 9 149–154. 10.1002/mrdd.10074 12953293

[B35] Moltesen M. Vindas M. A. Winberg S. Ebbesson L. de Lourdes Ruiz-Gomez M. Skov P. V. (2016). Cognitive appraisal of aversive stimulus differs between individuals with contrasting stress coping styles; evidences from selected rainbow trout (*Oncorhynchus mykiss*) strains. *Behaviour* 153 1567–1587. 10.1163/1568539X-00003405

[B36] Munakata A. Björnsson B. T. Jönsson E. Amano M. Ikuta K. Kitamura S. (2000). Post-release adaptation processes of hatchery-reared honmasu salmon parr. *J. Fish Biol.* 56 163–172. 10.1111/j.1095-8649.2000.tb02092.x

[B37] Navas J. M. Anglade I. Bailhache T. Pakdel F. Breton B. Jégo P. (1995). Do gonadotrophin-releasing hormone neurons express estrogen receptors in the rainbow trout? A double immunohistochemical study. *J. Comp. Neurol.* 363 461–474. 10.1002/cne.903630309 8847411

[B38] Northcutt R. G. (2006). Connections of the lateral and medial divisions of the goldfish telencephalic pallium. *J. Comp. Neurol.* 494 903–943. 10.1002/cne.20853 16385483

[B39] O’Connell L. A. Hofmann H. A. (2011). The vertebrate mesolimbic reward system and social behavior network: a comparative synthesis. *J. Comp. Neurol.* 519 3599–3639. 10.1002/cne.22735 21800319

[B40] Okuno H. (2011). Regulation and function of immediate-early genes in the brain: beyond neuronal activity markers. *Neurosci. Res.* 69 175–186. 10.1016/j.neures.2010.12.007 21163309

[B41] Olla B. L. Davis M. W. Ryer C. H. (1998). Understanding how the hatchery environment represses or promotes the development of behavioral survival skills. *Bull. Mar. Sci.* 62 531–550.

[B42] Palmé A. Wennerstöm L. Guban P. Ryman N. Laikre L. (2012). Compromising Baltic salmon genetic diversity - conservation genetic risks assiciated with compensatory releases of salmon in the Balctic Sea. *Havs Vattenmyndighetens Rapp.* 2012:115.

[B43] Pang P. T. Teng H. K. Zaitsev E. Woo N. T. Sakata K. Zhen S. (2004). Cleavage of proBDNF by tPA/plasmin is essential for long-term hippocampal plasticity. *Science* 306 487–491. 10.1126/science.1100135 15486301

[B44] Parrish D. L. Behnke R. J. Gephard S. R. McCormick S. D. Reeves G. H. (1998). Why aren’t there more Atlantic salmon (Salmo salar)? *Can. J. Fish. Aquat. Sci.* 55 281–287. 10.1139/cjfas-55-S1-281

[B45] Pavlidis M. Theodoridi A. Tsalafouta A. (2015). Neuroendocrine regulation of the stress response in adult zebrafish, *Danio rerio*. *Prog. Neuro Psychopharmacol. Biol. Psychiatry* 60 121–131. 10.1016/j.pnpbp.2015.02.014 25748166

[B46] Portavella M. Torres B. Salas C. (2004). Avoidance response in goldfish: emotional and temporal involvement of medial and lateral telencephalic pallium. *J. Neurosci.* 24 2335–2342. 10.1523/jneurosci.4930-03.2004 14999085 PMC6730421

[B47] Pottinger T. G. Pickering A. D. Hurley M. A. (1992). Consistency in the stress response of individuals of two strains of rainbow trout, *Oncorhynchus mykiss*. *Aquaculture* 103 275–289. 10.1016/0044-8486(92)90172-H

[B48] Rodrìguez F. López J. C. Vargas J. P. Gómez Y. Broglio C. Salas C. (2002). Conservation of spatial memory function in the pallial forebrain of reptiles and ray-finned fishes. *J. Neurosci.* 22 2894–2903. 11923454 10.1523/JNEUROSCI.22-07-02894.2002 PMC6758289

[B49] Rosen J. B. Fanselow M. S. Young S. L. Sitcoske M. Maren S. (1998). Immediate-early gene expression in the amygdala following footshock stress and contextual fear conditioning. *Brain Res.* 796 132–142. 10.1016/S0006-8993(98)00294-7 9689463

[B50] Rueden C. T. Schindelin J. Hiner M. C. DeZonia B. E. Walter A. E. Arena E. T. (2017). ImageJ2: ImageJ for the next generation of scientific image data. *BMC Bioinformatics* 18:529. 10.1186/s12859-017-1934-z 29187165 PMC5708080

[B51] Salonius K. Iwama G. K. (1993). Effects of early rearing environment on stress response, immune function, and disease resistance in juvenile coho (*Oncorhynchus kisutch*) and Chinook salmon (*O. tshawytscha)*. *Can. J. Fish. Aquat. Sci.* 50 759–766. 10.1139/f93-087

[B52] Schindelin J. Arganda-Carreras I. Frise E. Kaynig V. Longair M. Pietzsch T. (2012). Fiji: an open-source platform for biological-image analysis. *Nat. Methods* 9 676–682. 10.1038/nmeth.2019 22743772 PMC3855844

[B53] Shors T. J. Anderson M. L. Curlik D. M. Nokia M. S. (2012). Use it or lose it: how neurogenesis keeps the brain fit for learning. *Behav. Brain Res.* 227 450–458. 10.1016/j.bbr.2011.04.023 21536076 PMC3191246

[B54] Singewald G. M. Rjabokon A. Singewald N. Ebner K. (2011). The modulatory role of the lateral septum on neuroendocrine and behavioral stress responses. *Neuropsychopharmacology* 36 793–804. 10.1038/npp.2010.213 21160468 PMC3055728

[B55] Stewart A. M. Kalueff A. V. (2012). The developing utility of zebrafish models for cognitive enhancers research. *Curr. Neuropharmacol.* 10 263–271. 10.2174/157015912803217323 23449968 PMC3468880

[B56] Thorstad E. B. Forseth T. (2015). “Status for norske laksebestander i 2015,” in *Report of Vitenskapelig rå*d for lakseforvaltning (Nr. 8). Edinburgh: North Atlantic Salmon Conservation Organization, 300.

[B57] Thorstad E. B. Uglem I. Arechavala-Lopez P. Økland F. Finstad B. (2011). Low survival of hatchery-released Atlantic salmon smolts during initial river and fjord migration. *Boreal Environ. Res.* 16 115–120.

[B58] Tognoli C. Rossi F. Di Cola F. Baj G. Tongiorgi E. Terova G. (2010). Acute stress alters transcript expression pattern and reduces processing of proBDNF to mature BDNF in *Dicentrarchus labrax*. *BMC Neurosci.* 11:4. 10.1186/1471-2202-11-4 20074340 PMC2829032

[B59] Vargas J. P. López J. C. Portavella M. (2009). What are the functions of fish brain pallium? *Brain Res. Bull.* 79 436–440. 10.1016/j.brainresbull.2009.05.008 19463910

[B60] Vaynman S. Ying Z. Gomez-Pinilla F. (2004). Hippocampal BDNF mediates the efficacy of exercise on synaptic plasticity and cognition. *Eur. J. Neurosci.* 20 2580–2590. 10.1111/j.1460-9568.2004.03720.x 15548201

[B61] Vindas M. A. Fokos S. Pavlidis M. Höglund E. Dionysopoulou S. Ebbesson L. O. E. (2018). Early life stress induces long-term changes in limbic areas of a teleost fish: the role of catecholamine systems in stress coping. *Sci. Rep.* 8:5638. 10.1038/s41598-018-23950-x 29618742 PMC5884775

[B62] Vindas M. A. Gorissen M. Höglund E. Flik G. Tronci V. Damsgård B. (2017). How do individuals cope with stress? Behavioural, physiological and neuronal differences between proactive and reactive coping styles in fish. *J. Exp. Biol.* 220(Pt 8), 1524–1532. 10.1242/jeb.153213 28167808

[B63] Woodward C. C. Strange R. J. (1987). Physiological stress responses in wild and hatchery-reared rainbow trout. *Trans. Am. Fish. Soc.* 116 574–579.

