# Supplementary figures and images for: Neurobiology of Wild and Hatchery-Reared Atlantic Salmon: How Nurture Drives Neuroplasticity
Source: Front Behav Neurosci. 2018 Sep 11;12:210. doi: 10.3389/fnbeh.2018.00210 (PMC6141658; doi:10.3389/fnbeh.2018.00210)

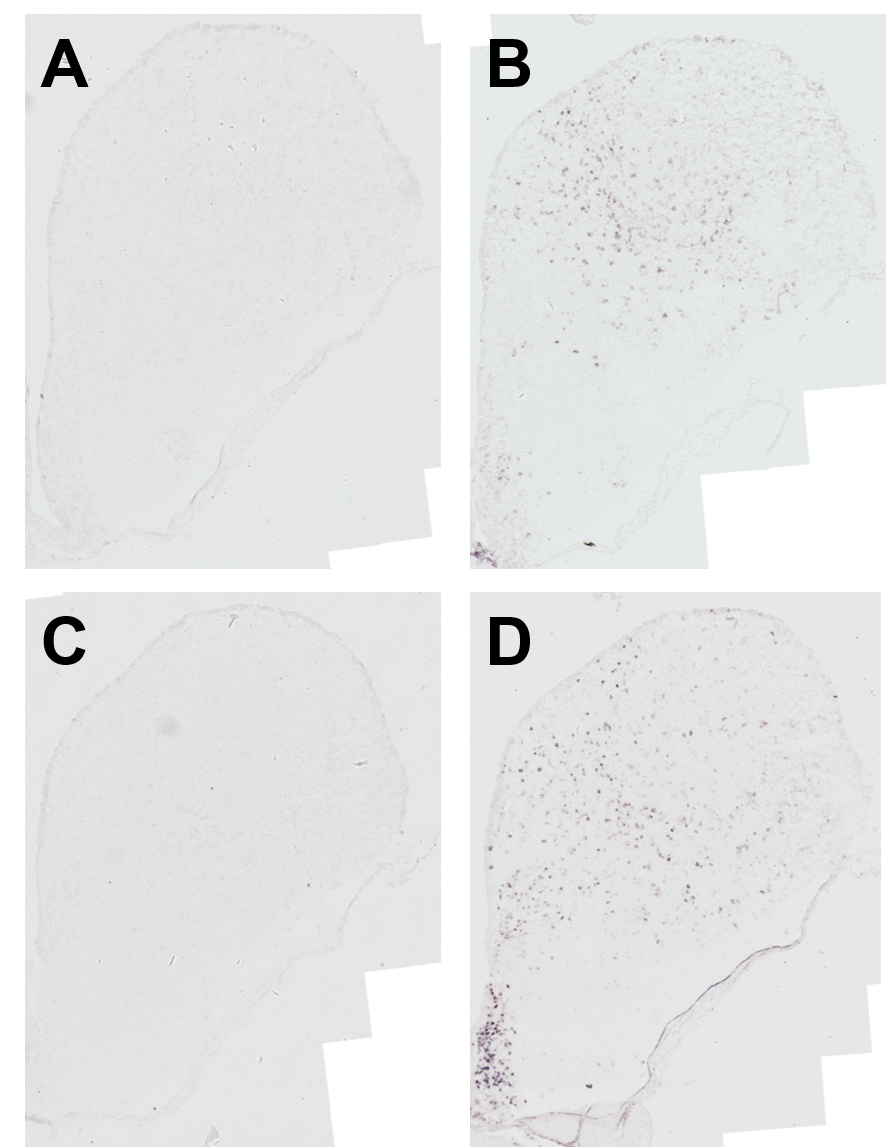

Supplement: FIGURE S1 — Specificity of the riboprobes was verified by performing the in situ hybridization protocol using both sense and antisense probes on a subset of brain samples. Depicted are the sense (A) and antisense (B) probes for bdnf and the sense (C) and antisense (D) probes for cfos. [file Image_1.TIF]

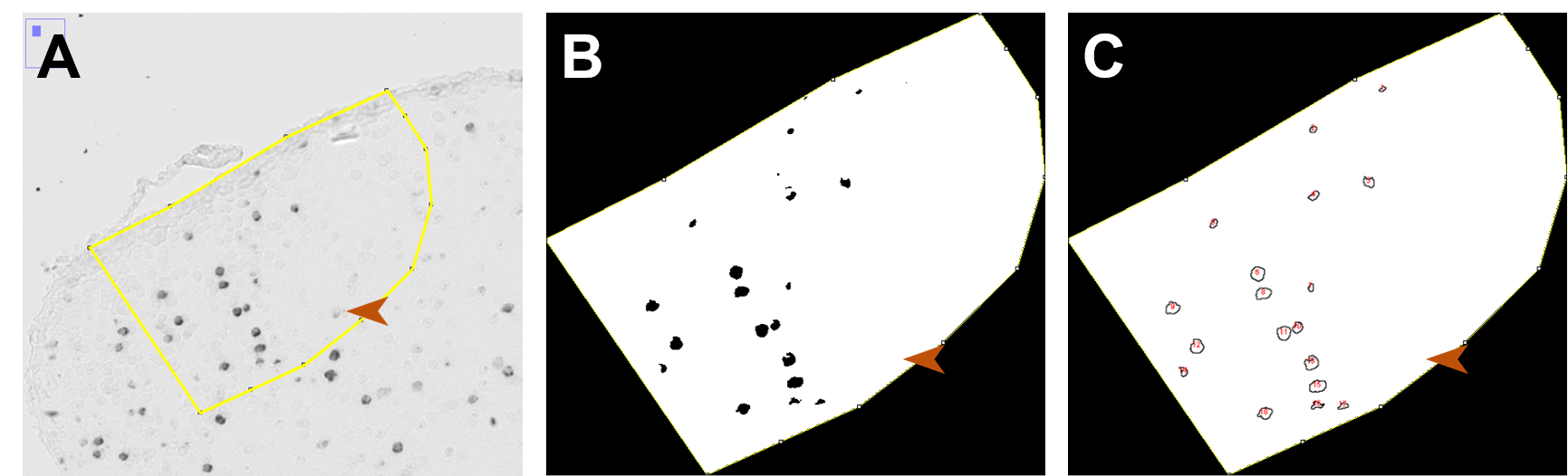

Supplement: FIGURE S2 — Illustration of several steps of the semi-automated quantification method of transcript-positive cells. The area of interest was selected using the selection tool (A), the black and white threshold was set to match the original image as closely as possible (B), and all cells that measures between 15 and 500 pixels were counted by the ImageJ software (C). Orange arrows indicate the location of a cell which is labeled too weakly to be quantified by the software after adjusting the black and white threshold (B). [file Image_2.TIF]
