# Supplementary material for: Neurobiology of Wild and Hatchery-Reared Atlantic Salmon: How Nurture Drives Neuroplasticity
Source: Front Behav Neurosci. 2018 Sep 11;12:210. doi: 10.3389/fnbeh.2018.00210 (PMC6141658; doi:10.3389/fnbeh.2018.00210)
Supplement: PRESENTATION S1 — Sequences of the riboprobes of bdnf and cfos used for in situ hybridization. [file Presentation_1.pdf]

Neurobiology of wild and hatchery-reared Atlantic salmon: how nurture drives neuroplasticity

Daan Mes, Kristine von Krogh, Marnix Gorissen, Ian Mayer, Marco A. Vindas

Supplementary File 1

Sequences of the riboprobes of *bdnf* and *cfos* used for *in situ* hybridization.

bdnf **(485 bp)**

tgcntGCTCgngCGGCCGCcaGTGTGATGGaTATCTGCAGAATTCGGCTTATGCCTCTTGT
CTATTCCACGGCAGCCCTCCTTTGTGTACCCCATAGGGTTACATTTGGTCTCATAAAA
GTATTGCTTCAGTTGGCCATTGGGGACAGGGACCTTTTCCAGGACGGTAACGGTCTG
CCCAGACATGTCTATTGCTGTCTTTTTGTCCACAGCTGTCACCCACTGGCTAATACTA
TCACACACACTCAGCTCTCCACGCCGCGACGGGTCAGAATGCCGCCGCACCCTCATG
GACATGTTAGCGGCGTCCAGGTAGTTTTTGTATTCCTCCAGGAGAAAAAGCAGCGGT
GGCTCTAAAGGCACTTGGTTGCTGATCATCACCCGCGATGCATACAGGTCGACATCC
TTGGTCTCCGTGGTGACCACAGAGGAAGGACCCCCTCCTCCCTGGCCCTTGTCAGCC
CCAGGCCCCAGCTGAGATGCTTCTCCTTCCACCTCCAAGAGCTCCTCAATCACCTGC
TCAAACGTGTCTGTGAAAGCCGAATTCCAGCACACTGGCGGCCGTTACTAGTGGATC
CGAGCTCGGTACCAAGCTTGGCGTAATCATGGTCATAGCTGTTTCCTGTGTGAAATT
GTTATCCGCTCACAATTCCACACAACATACGAGCCGGAAGCATAAAGTGTAAAGCC
TGGGGTGCCTAATGAGTGAGCTAACTCACATTAATTGCGTTGCGCTCACTGCCCGCT
TTCCAGTCGGGAAACCTGTCGTGCCAGCTGCATTAATGAATCGGCCAACGCGCGGG
GAGAGGCGGTTTGCGTATTGGgCGCTCTTCCGCTTCCTCGCTCACTGACTCGCTGCGC
TCGGTCGTTCGGCTGCGGCGAGCGGTATCAGCTCACTCAAAGGCGGTAATACGGTTA
TCCACAGAATCAGGGGaTAACGCAGGAAnGAACATGTGAGCAAAnGGCCAGCAAAA
GGCCAGGAACCGTAAAAAGGCCGCGTTGCTGGCGTTTTTCCATAGGCTCCGCCCCCC
TGACGAgCATCACAAAAaTCGACGCTCAAGTCagaGGTGGCGAAaCCcGaCAGGACTAT
AAagaTACCAGGCGTTtCCCccTGGAancTCCcTcGTGcgcTCTCCTGTTCcnaCCcTGCcgcTT
ACcGGatnCCTGTCcgCct

Primers used for cloning:

bdnf F1 ATGCCTCTTGTCTATTCCACGGCA

bdnf R1 TCACAGACACGTTTGAGCAGGTGA
Compl: TCACCTGCTCAAACGTGTCTGTGA

***cfos* (906 bp)**

NNNNNNNNNTACCCTNNCTAAAGGGACTAGTCCTGCAGGTTTAAACGAATTCGCCC
TTTGTAGAGAGGCTCCCAGTCCTGAGTGTACAGGGAGCTGGCCAGATCAACCTCGG
GCACCGACCGGGCCGTCTCCATCTCAGCCTTGGCGAGGAGAGACAGGGACTCCAGG
CAGGCTGTGTCCAGGTCGGCCATCTTGACGTCGGAGACGGAGATGGAGGCAGTGGA
CAGGAAGGGACTGCTGGAGAATATGGAGGCAGAGGCTGAGAGGGTGGAGGTGGAC
TGGATGGAAGTAAGGGGAGCGGCAGAGCAGGATACCATGCGCTGAGGCGGGGACA
GCTGGATGGAGACCCCGTGGGAGGGCGAGATGGAAGGGAAAGTCGTGTCCATATCA
GAGCGGATCTTGCAGATGGGCTGGTGGGCTGCCAAGATAAACTCTAACTTCTCCTTC
TCTTTAAGCAGGTTGGCGATGTCGTTCTGGAGAACAGACTTCTCTTCCTCCAGCTCGT
CGGTTTCACCCTGCAGAGTGTCGGTGAGTTCCTTTCGCCTGTTGCGGCATTTAGCTGC
TGCCTGCTTGTTCCTCTCTCTACGGACTCGCTTCTTCTCCTCCTCTTCAGGCGAAAGC
TGTTCCATTTTGCCTCTGCGCCCAGAGCTGTGGCCCTTGTTCCTCATGGCTCTAGTGT
AGGTTGGGGGGCTGTCGCTGTAGGGATGGGCTCTGTGAGAAGGTGCCACAGAGGAT
AGCGGCTGGACCAACCACTGCAGGTCTGGGCTGGCAGAGATGGCTGTAACAGTAGG
GATGAAGGACGGACCACTGGACACTGAATTCGGGTCTGTGAAGTCCTGAGATTGGG
GTGAACCCATGCTGGAGTAAGATCCCTCNNGAGAGTTGAAGTAAACCAGCTTGTCG
CNNNATGGAGAAGCTGTACTACAGCGAGAAGAANANTCACAGTCGGTGTTGAAAGC
GGAGTANNNNGAATTCGCGGCCGCTAAATTCAATTCGCCCTATANTGAGTCGTATTA
CNATTCACTGGNNGTCNTTTTACAACGTCNNGACTGGGAAAANNN

Primers used for cloning:

cFosF1: ACTCCGCTTTCAACACCGAC
Compl: GTCGGTGTTGAAAGCGGAGT

cFosR1: TGTAGAGAGGCTCCCAGTCC
