# Supplementary material for: Neurobiology of Wild and Hatchery-Reared Atlantic Salmon: How Nurture Drives Neuroplasticity
Source: Front Behav Neurosci. 2018 Sep 11;12:210. doi: 10.3389/fnbeh.2018.00210 (PMC6141658; doi:10.3389/fnbeh.2018.00210)
Supplement: PRESENTATION S2 — The macro script was used for quantification of labeled cells using the Fiji platform in ImageJ2. [file Presentation_2.pdf]

Neurobiology of wild and hatchery-reared Atlantic salmon: how nurture drives neuroplasticity

Daan Mes, Kristine von Krogh, Marnix Gorissen, Ian Mayer, Marco A. Vindas

Supplementary File 2

The following macro script was used for quantification of labeled cells using the Fiji platform in ImageJ2.

```
run("Rotate... ");
run("8-bit");
setTool("polygon");
waitForUser
run("Crop");
setBackgroundColor(0, 0, 0);
run("Clear Outside");
run("Threshold...")
waitForUser
run("Convert to Mask");
run("Fill Holes");
run("Watershed");
run("Analyze Particles...", "size=15-1000 pixel show=Outlines clear summarize");
```
